# Pediatric metabolic (dysfunction)-associated fatty liver disease: current insights and future perspectives

**DOI:** 10.1007/s12072-024-10691-5

**Published:** 2024-06-16

**Authors:** Sunitha Vimalesvaran, Pietro Vajro, Anil Dhawan

**Affiliations:** 1 Paediatric Liver, Gastroenterology and Nutrition Centres, King’s College Hospital NHS Trust, London, UK; 2 Department of Medicine, Surgery and Dentistry “Scuola Medica Salernitana”, Section of Pediatrics, Baronissi, Salerno Italy

**Keywords:** Metabolic (dysfunction)-associated fatty liver disease (MAFLD), Non-alcoholic fatty liver disease (NAFLD), Children, Adolescents, Fatty liver disease, Obesity, Gut microbiome, Lifestyle, Diet, Nutrition, Weight loss

## Abstract

The historical use of the term non-alcoholic fatty liver disease (NAFLD) in obese/overweight children has been controversial as to the appropriateness of this terminology in children, and lately, in adults too. Newer game-changer terminology, metabolic (dysfunction)-associated fatty liver disease (MAFLD), for this condition signifies a positive step forward that addresses the limitations of the previous definition for both adults and children. The prevalence of MAFLD has surged in tandem with the global rise in obesity rates, establishing itself as a predominant cause of chronic liver disease in both adult and pediatric populations. The adoption of the recently proposed nomenclature reflects a more encompassing comprehension of the disease and its etiology compared to its predecessor, NAFLD. Notably, the revised terminology facilitates the recognition of MAFLD as an autonomous condition while acknowledging the potential coexistence of other systemic fatty liver disorders. Particularly in children, this includes various paediatric-onset genetic and inherited metabolic disorders, necessitating thorough exclusion, especially in cases where weight loss interventions yield no improvement or in the absence of obesity. MAFLD presents as a multifaceted disorder; evidence suggests its origins lie in a complex interplay of nutritional, genetic, hormonal, and environmental factors. Despite advancements, current non-invasive diagnostic biomarkers exhibit limitations in accuracy, often necessitating imaging and histological evaluations for definitive diagnosis. While dietary and lifestyle modifications stand as cornerstone measures for MAFLD prevention and management, ongoing evaluation of therapeutic agents continues. This article provides an overview of the latest developments and emerging therapies in the realm of paediatric MAFLD.

## Introduction

The terms non-alcoholic fatty liver disease (NAFLD) and non-alcoholic steatohepatitis (NASH) have been in practice for the last 40 years [1]. The appropriateness of this terminology has recently been questioned particularly in children, as alcoholism in this population is rare and obesity may mask an underlying inherited metabolic defect affecting the liver like Wilson disease, with devastating consequences if left untreated [2, 3]. Pediatric experts have continued to lobby for the definition to be more inclusive and finally welcomed the game changer term metabolic (dysfunction)-associated fatty liver disease (MAFLD), based on a set of simple positive rather than negative diagnostic criteria, overarching and representative of this condition in children [4]**.**

Childhood obesity is a growing epidemic of the twenty-first century, affecting more than 340 million children and adolescents aged 5–19 years, according to data from the WHO [5]. This is no doubt of major concern, considering the association of obesity with other long-term health problems, such as cardiovascular disease, type 2 diabetes and cancer. Whilst these were rarely found in children previously, these diseases are increasingly being described in children and adolescents. Parallel to this rising trend in obesity, fatty liver disease has been increasingly recognised as the most common chronic liver disease at all ages. It represents an enormous global health concern. Depending on the definition used (i.e. whether screening by serum alanine aminotransferase (ALT) and ultrasonography or by liver biopsy), the prevalence of fatty liver disease in children varies. In the USA, the prevalence has nearly doubled; with 9.6% of all children between the ages 2 and 19 years and 38% of children with obesity estimated to have fatty liver disease (defined by being overweight with a body-mass index (BMI) > 95th percentile with high ALT concentrations) [6]. In the UK, 1 in 5 young people are estimated to have fatty liver disease with progression to fibrosis in 1 in 40, by the age of 24 years [7]. Studies from Asia suggest an overall prevalence of fatty liver disease in 5.5% of children with its pooled prevalence rate increasing to 50.1% in obese children [8]. Worldwide, the overall prevalence of fatty liver disease is estimated to be between 3–10% in the general pediatric population [9].

Over the past 5–10 years, developments in this field have revolutionized our understanding of the genetic factors, natural history, diagnostic modalities, and therapeutic options for this disease. In this review, we summarize the current knowledge and advances in the field of paediatric fatty liver disease.

Defining and diagnosing fatty liver disease in Children.

The landscape of fatty liver disease in children has rapidly evolved, with pediatric experts in the field having reservations about the term NAFLD due to the rarity of alcohol consumption in this cohort of patients and steatosis in liver as a non-specific marker of several inherited metabolic liver disorders. NAFLD was originally used to summarize a spectrum of liver disease, characterized by a histological diagnosis of fat accumulation in > 5% of hepatocytes, from simple steatosis to non-alcoholic steatohepatitis (NASH) with inflammation and fibrosis. It has also been used to define a pre-NASH state, characterized by tissue necroinflammation, hepatocellular injury and eventually fibrosis (non-alcoholic steatofibrosis) [10].

Increasing evidence has indicated that fatty liver disease is a hepatic manifestation of a systemic metabolic disorder, linking fatty liver disease to cardiometabolic derangements, even in childhood [11]. With the increasing knowledge of the dominant role of metabolic dysfunction in this patient cohort, an international expert consensus panel proposed metabolic (dysfunction)-associated fatty liver disease (MAFLD) as the new name for NAFLD [12]. The proposed new term is more than a semantic revision but underpins the integral link with a metabolic milieu [13, 14]. It allows for the disease to be managed as a stand-alone disease while acknowledging the possibility of other coexisting conditions with fatty liver disease, and avoids the stigmatization associated with the word ‘alcoholic’ present in the term NAFLD. It also avoids the term obesity but stresses the importance of metabolic dysfunction in its pathogenesis. Overall, this change allows for a more comprehensive understanding of the disease and its underlying causes.

In adults, the diagnostic criteria for MAFLD are based on the radiological evidence of hepatic steatosis and the presence of one of three criteria: overweight or obesity, type 2 diabetes, or evidence of metabolic dysfunction [12]. The morbid state of fatty liver disease is more heterogeneous in children, which can be sub-classified as follows [3]: type 1, fatty liver with an inherited metabolic defect such as defects involving carbohydrate, protein and lipid metabolism, mitochondrial disorders and aminoacyl tRNA synthetase disorders; type 2, fatty liver associated with metabolic dysfunction (MAFLD); or type 3, fatty liver in those who do not fulfill the criteria for metabolic dysfunction and should be considered separately, bearing in mind the possibility of identifying a yet undiagnosed inherited metabolic defect. The main focus of this review will be on type 2 paediatric fatty liver disease or MAFLD, the most prevalent type of fatty liver disease.

**Fig. 1:**
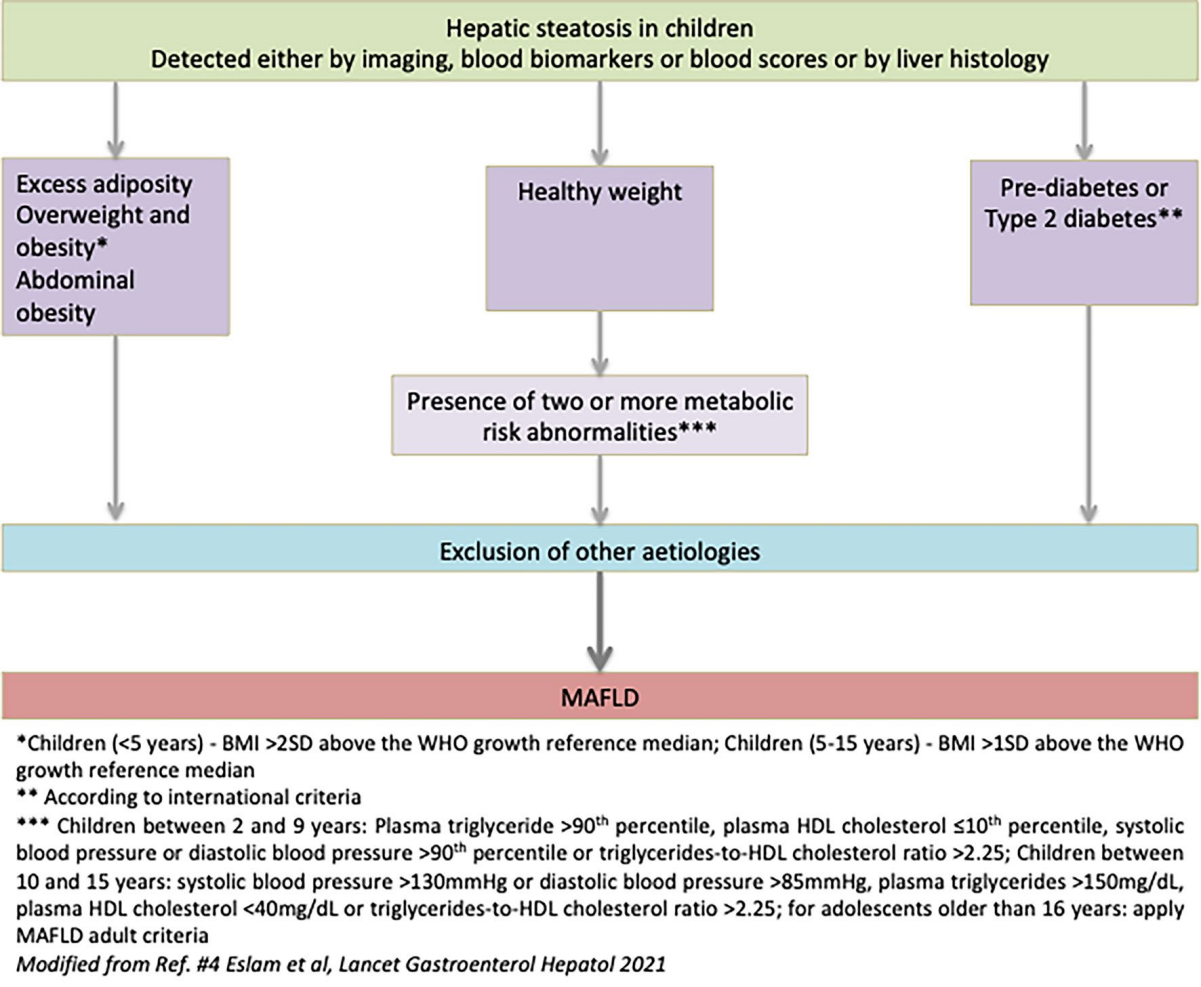
Proposed diagnostic criteria (Modified from Ref. #4 Eslam et al, Lancet Gastroenterol Hepatol 2021)

The recent proposed diagnostic criteria, through an international consensus for paediatric MAFLD, are based on liver histology, imaging and/or blood biomarker evidence of intrahepatic fat accumulation, in addition to one of the three criteria: excess adiposity, presence of prediabetes or type 2 diabetes, or evidence of metabolic dysregulation (Fig. 1) [4]. It is important to recognise conditions that can be associated with MAFLD but also those that can mimic it; in particular a sub-category of subjects—lean patients with MAFLD due to inborn errors of metabolism [15]. As such, MAFLD in children is probably best defined as *chronic hepatic steatosis in children*, with excess adiposity or other evidence of metabolic dysfunction such as type 2 diabetes and after exclusion of alternative diagnoses such as inherited metabolic disorders, infections, use of steatogenic medications, ethanol consumption and malnutrition.

### Etiology

Over the past decade, developments in this field have greatly improved our understanding of genetic predisposition, antenatal maternal health, natural history, diagnostic modalities, and therapeutic targets for this disease. A complex interplay of nutritional, genetic and epigenetic factors is thought to contribute to MAFLD in children.

#### Diet and nutrition

**Fig. 2:**
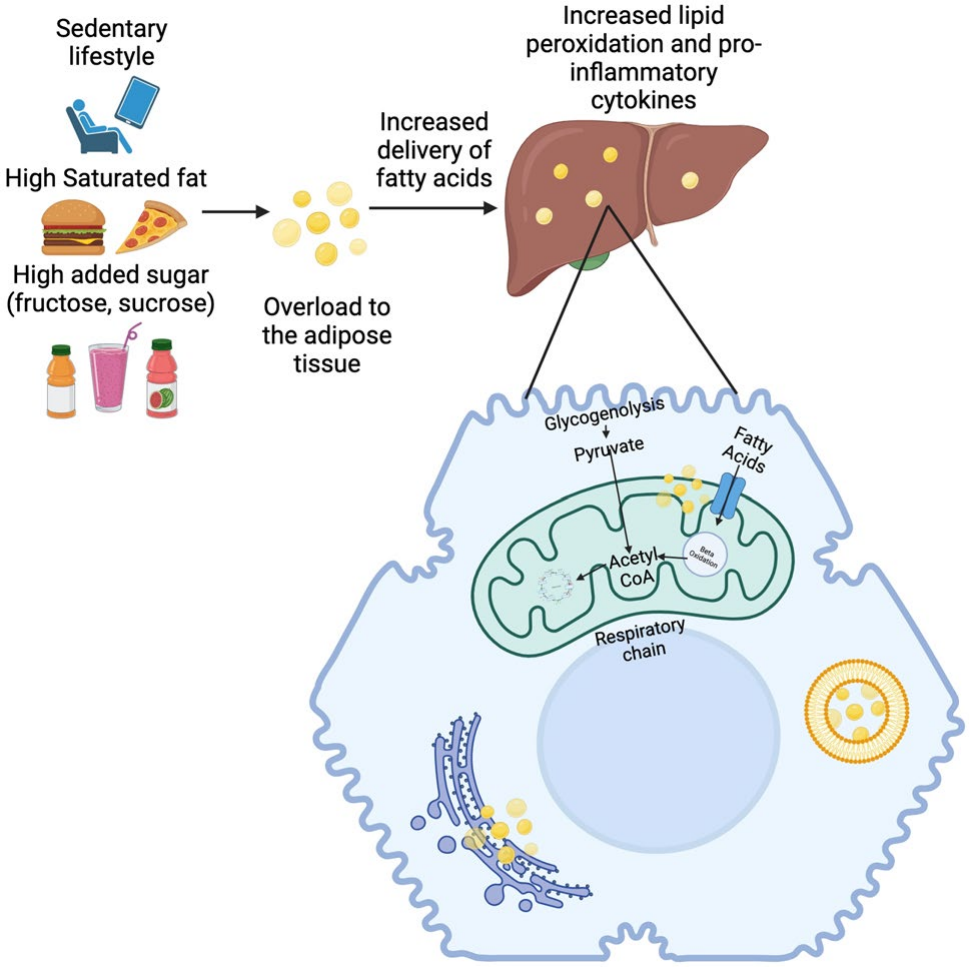
Diets high in saturated fats, sucrose and fructose are steatogenic and cause dysregulation of key lipid metabolic pathways and hormones. The development of insulin resistance in MAFLD leads to increased adipocyte lipolysis and high circulating free fatty acids available for subsequent hepatic uptake. This leads to intrahepatic lipid accumulation. In hepatocytes, the inability to accommodate neutral lipids within lipid droplets exposes cells to lipotoxic bioactive lipids. Lipotoxicity causes oxidative damage and promotes inflammation and fibrosis through a number of mechanisms. (Created with Biorender)

The link between increased BMI and risk of developing MALFD in children and adults has been well elucidated (Fig. 2). The 2009–2012 National Health and Nutrition Examination Survey showed that 13–17% of calories in the diet of American children composed of sugar added to food and beverages [16]. Free sugar consumption has a significant role in promoting insulin resistance, hyperuricemia and the development of MAFLD. Many research groups have sought to establish the role of free sugar and fructose intake and the development of obesity and MAFLD in the paediatric population [17, 18]. The quality of fat in the diet also plays an important role in the development of MAFLD [19–21]. Consumption of an isocaloric diet made of saturated fatty acids can contribute to hepatic fat accumulation, as opposed to diets high in polyunsaturated fatty acids (PUFA), especially omega-3 fatty acids [22]. With this in mind, dietary habits, and nutrition from early life, are likely to have a significant role in the development of MAFLD in children.

#### Gut Microbiome

Increasingly dysbiosis has also been an area of interest. The “two hit” hypothesis was first suggested by Day and James [23] with the initial insult resulting in the development of hepatic steatosis, whilst the second involves oxidative stress and lipid peroxidation, ultimately causing steatohepatitis. Recently, the gut microbiome (GM) has been implicated in each of these multiple “hits”, capable of triggering and exacerbating MAFLD pathophysiology [24, 25]. 

**Fig. 3:**
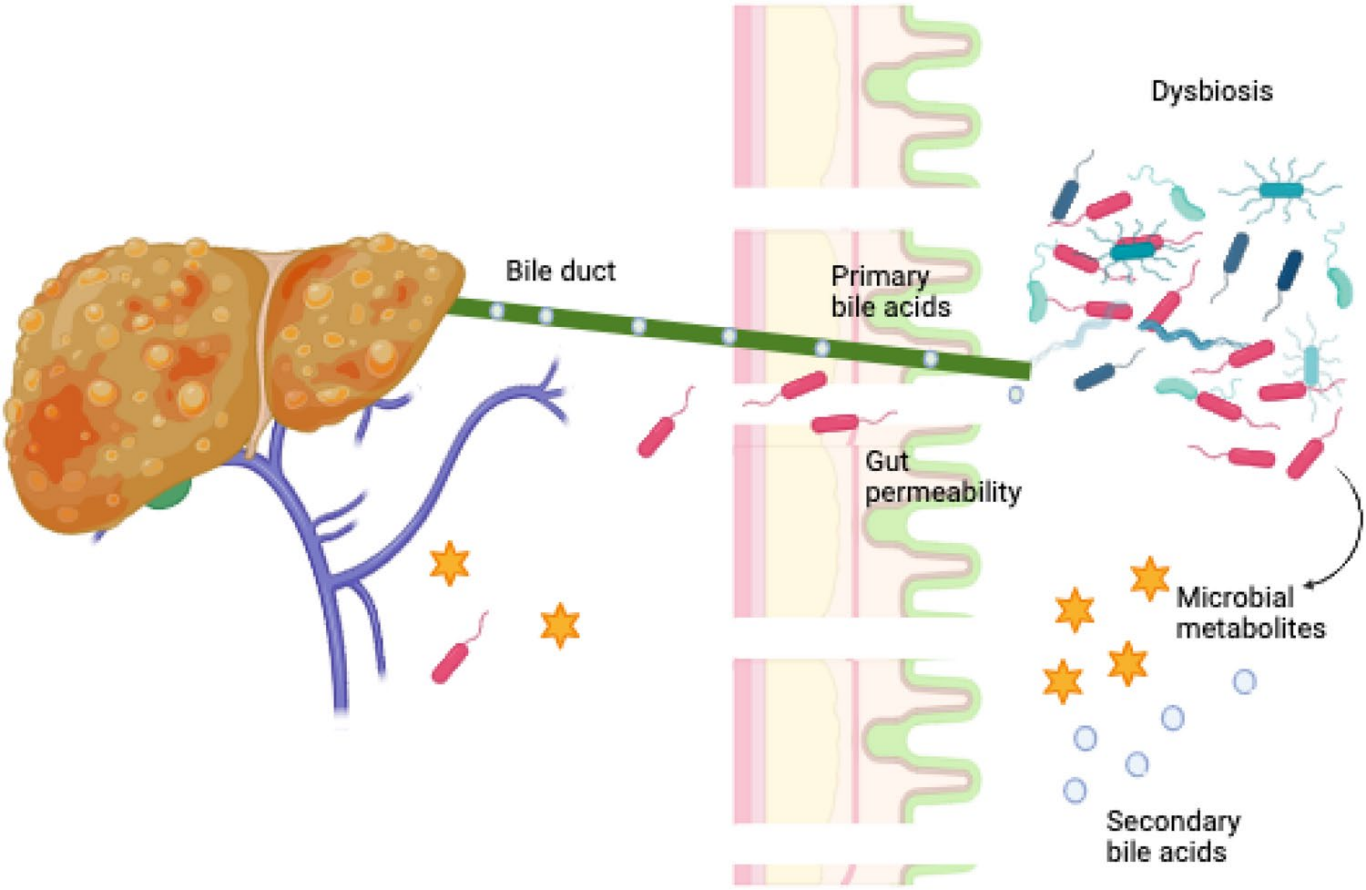
The intestinal barrier is composed of a mucus layer, intestinal epithelium, mucosal immune system, and gut vascular barrier. When its integrity is damaged by an abnormal GM composition (gut dysbiosis) there is an increased intestinal permeability leading to unrestrained transfer of noxious bacterial substances/metabolites (SCFA, ethanol, LPS) and various pro-inflammatory molecules to the liver through the portal system (Gut-Liver Axis). These events contribute to steatosis and accelerate inflammatory and fibrogenic progression of MAFLD. Moreover bile acids (BA) produced by hepatocytes are further metabolized by a dysbiotic GM and may result in an atypical primary/secondary BA pool (DCA > CDCA). This a) interferes with Tight Junctions, and b) binds atypically to the BA receptors (e.g. TGR5, FXR and VDR) along the enterohepatic system, resulting in carbohydrate and lipid metabolism dysfunction [1]. (Created with Biorender). *GM* Gut Microbiome, *SCFA* Short chain fatty acid, *LPS* lipopolysaccharide, *BA* Bile acids, *DCA* deoxycholic acid, *CDCA* chenodeoxycholic acid, *TGR5* Takeda G protein receptor 5, *FXR* Farnesoid X receptor, *VDR* vitamin D receptor

Murine models have provided significant evidence for the GM in obesity. Germ-free (GF) mice showed resistance to developing obesity in the absence of intestinal bacteria [26]. Later on, faecal microbiota transplant from faecal samples of mice with obesity resulted in obesity in recipient GF mice, while transfer of samples from lean individuals did not [27]. This was also associated with increased systemic inflammation and metabolic syndrome. Mouse models of steatosis and MAFLD, and preliminary clinical and experimental studies both in adults and in children have shown compelling evidence that the dysfunction of the so-called gut-liver axis (GLA) [i.e. intestinal dysbiosis, small intestinal bacterial overgrowth (SIBO), and increased intestinal permeability to bacterial products (leaky gut)] is a major factor in the development and progression of MAFLD [28–33] (Fig. 3).

In paediatrics, a number of studies have thus far studied the relationship between the intestinal microbiome in children with MAFLD [34–38] (Table 1). All studies consistently show that MAFLD is associated with dysbiosis and loss of alpha-diversity of the intestinal microbiome. The studies thus far are limited by differences in study design and geographic and ethnic differences, which results in poor comparability and inability to draw firm conclusions in relation to bacterial composition. A number of controversies such as the involvement of microbial endogenous alcohol production and the importance of the abundance of specific bacterial taxa, such as *Prevotella* and *Escherichia* remain [39].

**Table 1:** Studies on the relationship between the intestinal microbiome in children with MAFLD

Study	Country	Study population	Microbial diversity		Microbial abundance	
			Alpha	Beta	Phylum	Genu/species
Stanislawski et al. [35]	US	8 NAFLD 99 Control	Decreased	Differed	Lower Bacteroidetes Lower B:F ratio	↑Paraprevotella ↑RF32 ↑Sutterella ↑Bilophila ↓Varibaculum ↓Oscillospira
Schwimmer et al. [34]	US	87 NAFLD 37 Control	Decreased	No difference	Increased Bacteroidetes Increased Proteobacteria Decreased Firmicutes	↑Prevotella copri
Zhu et al. [38]	US	22 NASH 25 obese 16 control	Decreased	Differed	Increased Bacteroidetes Increased Proteobacteria Decreased Firmicutes	↑Prevotella ↑Escherichia ↓Alistipes ↓Blautia ↓Coprococcus ↓Eubacterium ↓Oscillospira ↓Bifidobacterium
Del Cherico et al. [36]	Italy	27 NAFLD 26 NASH 8 obese 54 control	Decreased	Differed	Decreased Bacteroidetes Increased Actinobacteria	↑Dorea ↑Ruminococcus ↓Oscillospira
Zhao et al. [40]	China	25 obese + NAFLD 18 obese 15 Control	Not significant	NA	Increased Proteobacteria Decreased Bacteroidetes	↑Phascolarctobacterium ↓Lactobacillus ↓Oscillibacter ↓Ruminiclostridium

*B: F* Bacteroidetes: Firmicutes, *NAFLD* Non-alcoholic fatty liver disease, *NASH* Non-alcoholic steatohepatitis, *NA* Not applicable

#### Genetics and Epigenetics and Maternal Factors

A number of single nucleotide polymorphisms (SNPs) have been demonstrated to increase susceptibility to fatty liver [10] (Table 2). These variants have also been associated with significantly higher risks of cirrhosis and hepatocellular carcinoma (HCC). Other protective variants such as hydroxysteroid 17-beta dehydrogenase 13 (*HSD17B13*) TA splice variant rs72613567, mitochondrial amidoxime reducing component 1 (*MTARC1*) and *HSD17B13* have been found to be associated with a milder form of MAFLD in children. A combination of these susceptibility and protective alleles contributes to an overarching genetic component or risk score (GenComp), which may be considered the “first hit” [41].

**Table 2:** Single nucleotide polymorphisms (SNPs) have been demonstrated to increase susceptibility to fatty liver

Gene polymorphism	Function of gene	MAFLD association
Patatin-like phospholipase domain-containing protein 3 (*PNPLA3*) Iso148Met (rs738409)	Triacylglyerol lipase acylglycerol transacylase	Increased NAFLD risk and severity; highest prevalence in Mexican Americans
Transmembrane 6 superfamily member 2 (*TM6SF2*) Glu167Lys (rs58542926)	Unknown	Increased ALT, AST and hepatic fat
Glucokinase regulator (*GCKR*) Pro446Leu (rs1260326)	Glucokinase inhibitor	Hepatic steatosis, increased ALT
Membrane bound o-acyltransferase domain containing 7 (*MBOAT7*) Gly17Glu (rs641738)	Encodes lysophospholipid acyltransferase enzyme	Increased hepatic fat content, increased risk of fibrosis

Studies in children are still limited. The Non-alcoholic Steatohepatitis Clinical Research Network conducted a study involving 234 Hispanic boys and identified SNPs associated with the NAFLD activity score (NAS) on chromosome 8 in the trafficking protein particle complex 9 (TRAPPC9) as well as a region close to actin-related protein 5 associated with fibrosis. They also found 10 SNPs associated with obesity and 9 with insulin resistance, HbA1C and HOMA-IR [42]. Lee et al. found *PNPLA3* (rs738409), *TM6SF2* (rs58542926) and *SAMM50* (rs2073080 and rs3761472) to be independent risk factors in 228 children with fatty liver [43].

Incorporating these genetic risk alleles into clinical practice will be critical as new and emerging evidence in this field come to light. By identifying at-risk individuals early, the opportunity for early intervention will be imperative. Whether these risk alleles play a role in terms of disease progression, histological severity or age of onset will need further investigation [44].

The perinatal period has been suggested to have a critical impact on the development of obesity [45] and obesity-related conditions such as cardiometabolic diseases and MAFLD [46–48]. Studies have suggested exposure to maternal over-nutrition or undernutrition increases susceptibility to MAFLD in childhood and hastens progression to NASH across the lifespan, especially when offspring are exposed in the postnatal period to a high-fat (Western-style) diet [47]. Furthermore, maternal obesity and insulin resistance acting through the placenta lead to the intrauterine exposure of increased fetal insulin, lipids, inflammation and possibly hypoxia, which cause developmental programming of hepatosteatosis before birth [49]. However, the actual mechanisms by which the exposure to an adverse in-utero environment and the development of metabolic disease later in life remains largely undefined. There remains an urgent need to highlight early maternal and infant epigenetic, inflammatory and microbial markers that may predispose to metabolic disease and MAFLD.

### Diagnostic tools in paediatric MAFLD

The true burden of paediatric MAFLD and its progression to end-stage liver disease in adulthood is now increasingly recognized. MAFLD (term NASH was used in this report) can progress rapidly to advanced fibrosis and cirrhosis highlighting the importance of early diagnosis [50].

The hallmark of diagnosis of fatty liver is through the detection of steatosis usually by imaging techniques. Ultrasound is often the first – choice modality in view of its wider availability, however, this modality suffers from poor sensitivity of detecting steatosis less than 20%, inter-observer variability and distinguishing steatosis from steatohepatitis or fibrosis, and may not be accurate, particularly in children and must be interpreted with caution [51].

Non-invasive imaging tests, such as transient elastography (TE) are increasingly being used in the assessment of hepatic steatosis and fibrosis. Detection of 10–30% steatosis is possible alongside fibrosis using controlled attenuation pressure (CAP), based on ultrasound signals acquired by TE using the principle of attenuation of ultrasound signal through fat. Its use in adults has been validated for over a decade in fatty liver disease. In children, one cross-sectional study found a difference in CAP between patients with steatosis and those without and was able to grade different severities of steatosis [52]. Optimal cut-off values for children are yet to be determined but one study suggested an upper limit of normal at 248 dB/m for healthy children. However, no comparison was made to histological diagnosis or other causes of fatty liver disease. Whilst TE remains a promising modality for the assessment of MAFLD in children, there remain limitations due to operator dependence, probe size and skin-capsular distance [53].

Magnetic resonance (MR) imaging has shown high diagnostic accuracy [54]. Middleton et al., demonstrated that MR-estimated liver proton density fat fraction (PDFF) correlated well with histological steatosis grades in a large paediatric study [55]. Limitations of MR spectroscopy and MR elastography include need for deep sedation or general anesthesia and the lack of easy availability. Therefore, it is typically restricted for use in research settings. Validation of these modalities will be pivotal before they are fully integrated into clinical practice.

Histological evidence of hepatic steatosis with inflammation, with or without ballooning injury to hepatocytes and fibrosis, remains the gold standard for the diagnosis of MAFLD. Grading is done according to the proportion of hepatocytes containing fat macrovesicles on hematoxylin and eosin (H&E) staining (grade 0, < 5%; grade 1, 5–33%; grade 2, 34–66%; and grade 3, > 66%). The inflammation in children is more often portal-based, steatosis may be peri-portal in distribution, while ballooning is generally uncommon [56]. Subtle differences in histological findings between adults and children suggest different disease processes or reflect the potential differences in the maturing process between adults and children. It is difficult to quantify these snapshot findings in an evolving chronic process and the jury is still out regarding how often and in whom biopsies should be done to monitor disease progression, especially in children. Furthermore, histological specimens are susceptible to sampling error and inter-observer variability [57]. The North American guidelines (NASPGHAN) suggest performing a liver biopsy in patients with increased risk of NASH and/or advanced fibrosis such as those with high ALT levels (> 80 U/L), splenomegaly, or an aspartate aminotransferase (AST) to ALT ratio of > 1 [58]. Goyal et al. showed that in children undergoing assessment for fatty liver, there was a high prevalence of advanced disease at presentation with 10–25% having advanced fibrosis at initial presentation and 25–50% demonstrating steatohepatitis [59]. Histological examination is particularly important in cases where alternative or coexisting diagnoses may be relevant in a child [15, 60].

#### Biomarkers

Noninvasive assessment of MAFLD severity is extremely challenging due to the lack of specific biomarkers that can be used in lieu of liver biopsy. Those hitherto proposed include both individual circulating molecules and the use of composite algorithms derived from their combination. Among those of the former group which have been better studied in the paediatric context, adiponectin is an adipokine that has been found to have strong MAFLD diagnostic accuracy while several interleukins (e.g., IL-1, IL-6, and IL-17), have been proposed as inflammatory markers with quite strong diagnostic accuracy, i.e. with AUROC values exceeding 0.90. Select serological biomarkers (e.g. type III procollagen peptide (PIIINP) and hyaluronic acid (HA)) perform well in predicting advanced fibrosis (AUROCs > 0.90) but not mild-moderate fibrosis.

Composite algorithms combining multiple serological biomarkers may provide scores that are promising tools to predict MAFLD, fibrosis and NASH often borrowed from adult experience (e.g., APRI: AST to platelet ratio index). Some scores however have been developed specifically for children (e.g. the pediatric NAFLD fibrosis index (PNFI) and pediatric NAFLD fibrosis score (PNFS) [61]. PNFI demonstrated an area under the Receiver Operating Characteristics curve (AUROC) of 0.663 in children with significant fibrosis (stage 2 to 3) and was significant in differentiating children with hepatic fibrosis from children without fibrosis [62]. Meanwhile, Alkhouri et al. showed that a cutoff of 26% for the PNFS model would provide specificity, sensitivity, positive and negative predictive values of 92%, 31%, 41% and 88%, respectively, for predicting advanced fibrosis [63]. However, these studies are still limited by selection bias and relatively small sample sizes, and hence, merit further validation.

Metabolic signatures in the blood (arginine, glycine, and acylcarnitine (AC) AC5:1) [64] and also in other more accessible biological fluids (saliva – de novo fatty acid biosynthesis; saturated fatty acid beta-oxidation; butanoate metabolism; glycolysis and gluconeogenesis; tricarboxylic acid cycle; urea cycle; metabolism of proline, glutamate, aspartate and asparagine; valine, leucine and isoleucine (BCAA) degradation; amino-sugar metabolism; purine metabolism; and glycerophospholipid metabolism [65]/urine – glucose/1-methylhistidine, xylitol, phenylacetic acid and hydroquinone [66]) appear promising biomarkers as noninvasive markers to identify at-risk individuals early.

## Therapeutic landscape

The therapeutic landscape is developing rapidly with the identification of newer compounds in adults that can modify liver steatosis, inflammation and eventually fibrosis, with the hope that these new pharmacologic agents will become available for the paediatric population soon.

### Lifestyle and Dietary Modifications

Lifestyle modifications remain the mainstay of treatment. This includes teaching children to adopt a healthy lifestyle, and encouraging them to include a scheduled and supervised physical activity program into a daily routine. A number of randomized controlled trials (RCTs) have investigated the impact of dietary and lifestyle interventions in children with MAFLD [67]. Chan et al. conducted an RCT in 52 Chinese adolescents with obesity, evaluating the impact of a lifestyle intervention, counseling participants once a week for four months on diet and exercise followed by a maintenance phase (twice a month) for 52 weeks. They showed a reduction in body fat and intrahepatic triglyceride content on proton magnetic resonance spectroscopy [68]. Malecki et al. investigated the impact of a Mediterranean diet and an aerobic physical exercise routine on 49 patients between 3 and 16 years of age diagnosed with MAFLD on ultrasound. They were also limited to using electronic devices for less than 2 h a day. The results showed a decrease in AST and ALT in all patients including those who did not decrease their BMI [69]. A Danish study investigated the impact of a hypocaloric diet (three meals daily) and exercise for 1 h a day in 117 children with obesity (43% of whom had MAFLD and 50% who showed elevated AST and ALT and decreased insulin sensitivity). Data showed a decrease in body weight, an increase in insulin sensitivity and an improvement in MAFLD parameters including liver echogenicity and texture and decreased ALT [70].

Most studies have included interventions that entail the prescription of lifestyle changes (healthy diet and exercise) and thus must be considered that specific nutritional interventions have not been isolated from habit modifications. Whilst metabolic alterations associated with NAFLD are significantly reduced, these interventions also do not achieve a complete reversal of disease. Any dietary intervention must also ensure adequate nutrition and energy for growth in the specific age groups [67].

### Pharmacotherapy

At present, there are no approved therapies for MAFLD in children. Studies on pharmacologic agents in paediatric MAFLD remain sparse. As the number of adolescents transitioning to adult services with chronic liver disease secondary to MAFLD increase, we may face a growing need for liver transplantation due to cirrhosis and hepatocellular carcinoma.

Whilst lifestyle interventions are first-line, the rising incidence of MAFLD brings to question if alternative pharmacologic agents can be trialled in the paediatric age group early, if lifestyle interventions are not adhered to or if they do not result in significant improvement of the disease course.

In view of its antioxidant properties, Lavine et al. compared Vitamin E to metformin and a control group in a randomised, double-blind study of 173 patients between the ages of 8 and 17 years with biopsy-confirmed MAFLD. Over 96 weeks, participants were given daily doses of 800 IU of vitamin E, 1000 mg of metformin, or placebo. The results showed that neither vitamin E nor metformin were superior to placebo in attaining the primary outcome of sustained reduction in ALT level [71]. Vitamin E however, did show some benefit in terms of improvements in MAFLD activity, histology, including ballooning of hepatocytes, and the proportion that resolved steatohepatitis at 96 weeks. The American Association for the Study of Liver Diseases (AASLD) advises consideration of the use of vitamin E in children with the risks and benefits explained; metformin at 500 mg twice daily is not recommended specifically to treat fatty liver, but higher doses and its effects on fatty liver and metabolic dysfunction should be further evaluated.

In view of the dysbiosis of the gut microbiome in MAFLD, probiotics have also been explored as a therapeutic target. A meta-analysis of four paediatric studies by Gkiourtzis suggested that probiotic supplementation, particularly *Lactobacillus acidophilus* in combination with other strains of *Bifidobacterium* or *Lactobacillus,* may be beneficial in the improvement of AST and ALT, lipid parameter levels, ultrasonographic, and anthropometric characteristics in children with MAFLD. The impact on histological assessment of paediatric MAFLD needs further examination and the exact beneficial strain most useful in this cohort is yet to be determined [72]. Similar results were confirmed by another meta-analysis, which also included two symbiotic/prebiotic studies [73].

Omega-3 fatty acids such as docosahexaenoic acid (DHA) and eicosapentaenoic acid are recommended in adults for hypertriglyceridemia. A meta-analysis of use of omega-3 fatty acids in fatty liver disease resulted in a statistically significant reduction in transaminases concentration and significant improvement in liver steatosis assessed by ultrasonography and a decrease in BMI [74].

Bile acid (BA) metabolism and signalling derangement in MAFLD has been the target of several studies focusing on the natural farnesoid X receptor (FXR) antagonist ursodeoxycholic acid (UDCA). Data shows that in adults with NASH, UDCA effectively reduces serum levels of liver enzymes but has no significant effects on liver histology [75]. Unfortunately, in children UDCA appears largely unsatisfactory [76].

More recent studies investigating the semisynthetic FXR agonist, Obeticholic acid (OCA), conducted exclusively in adults with NASH, showed reduction in insulin resistance, steatohepatitis, levels of ALT and fibrosis in these patients. Its dose-related adverse effects (e.g. pruritus and dyslipidaemia) however may limit its usage [77].

Metabolic bariatric surgery has become a safer option in adolescents with morbid obesity, achieving improvement or even resolution of serious metabolic comorbidities including liver disease. It is therefore developing from a controversial topic to a viable strategic therapeutic component of various medical societies to possibly avoid end-organ damage in adulthood [78, 79].

### Perspectives and future directions

New anti-diabetic drugs like glucagon-like peptide 1 (GLP-1) agonists acting as anti-obesity drugs, have shown promising results in adults with MAFLD to reduce hepatic steatosis and liver enzyme activity [80]. Studies are yet to demonstrate if these agents are as promising in paediatric MAFLD patients.

Finally, modulation of the gut microbiome by allogenic faecal microbiota transplantation has been shown to restore intestinal permeability, improve hepatic steatosis and inflammation in murine models. Use in patients with MAFLD still remains highly experimental and has several safety limitations [81]. Data in the paediatric age group remains extremely scarce [82].

## Conclusion

MAFLD is increasingly becoming a major challenge to global public health. Whilst paediatric MAFLD shares many features in common with adults, there are key differences including early life influences, susceptibilities, approach to diagnosis and management, and additional differential diagnoses of possibly stand-alone or accompanying inherited metabolic defects that require specific disease-related therapies. 

With new discoveries in gene polymorphisms and the influence of the gut microbiome, there are real opportunities to institute MAFLD preventative measures and divert the course of the disease in childhood avoiding end-stage liver disease in adulthood.
